# Directed Kinetic Self-Assembly of Mounds on Patterned GaAs (001): Tunable Arrangement, Pattern Amplification and Self-Limiting Growth

**DOI:** 10.3390/nano4020344

**Published:** 2014-05-12

**Authors:** Chuan-Fu Lin, Hung-Chih Kan, Subramaniam Kanakaraju, Christopher Richardson, Raymond Phaneuf

**Affiliations:** 1 Department of Materials Science and Engineering, University of Maryland, College Park, MD 20742, USA; E-Mails: cflin@umd.edu (C.-F.L.); phyhck@ccu.edu.tw (H.-C.K.); 2 Laboratory for Physical Science, 8050 Greenmead Drive, College Park, MD 20740, USA; E-Mails: raju.subramaniam@epiworks.com (S.K.); richardson@lps.umd.edu (C.R.); 3 Department of Physics, National Chung-Cheng University, Chia-Yi 62102, Taiwan

**Keywords:** directed self-assembly, self-assembly, self-limiting behavior, pattern amplification, nanostructures, growth mounds, crystal growth

## Abstract

We present results demonstrating directed self-assembly of nanometer-scale mounds during molecular beam epitaxial growth on patterned GaAs (001) surfaces. The mound arrangement is tunable via the growth temperature, with an inverse spacing or spatial frequency which can exceed that of the features of the template. We find that the range of film thickness over which particular mound arrangements persist is finite, due to an evolution of the shape of the mounds which causes their growth to self-limit. A difference in the film thickness at which mounds at different sites self-limit provides a means by which different arrangements can be produced.

## 1. Introduction

One of the most daunting challenges posed by nanotechnology is achieving the fabrication of extremely high densities of nanometer-scale clusters of atoms, with positional control, and on a practical time scale. A seemingly attractive approach toward meeting this challenge involves the use of a template to direct the self-assembly of nanostructures [1,2,3,4,5,6,7,8]. However, only if assemblies result in which the individual structures are more complex than, or arrange at higher spatial frequencies than the features of the original template does this offer any advantage over conventional, “top down” fabrication. Higher feature spatial frequencies, or “pattern amplification” [9,10,11,12] can result if the wavelength selection displayed in certain growth instabilities can be anchored by features of a template of lower spatial density. Understanding how an artificially defined template interacts with such instabilities could enable control of the positions and densities of spontaneously forming nanostructures. In this letter we examine the effect of an artificially imposed template in a simple system known to display a type of self-assembly: that of nanometer scale multilayer islands, or “mounds”, which form during homoepitaxial growth on GaAs (001). A key difference between this system and those in which template-directed assembly of multilayer islands (“quantum dots”) have previously studied, including Ge on patterned Si (001) [4,5] and InAs on patterned GaAs (001) [6,7] is the absence of strain as a driving force for self-assembly [8]. Instead mound formation during homoepitaxy is generally thought to be entirely due kinetics, in particular to an instability [13] resulting from an extra (“Ehrlich-Schwoebel”) barrier to the diffusion of atoms across atomic steps from above [14,15,16,17]. We find that a predefined topographical pattern on a GaAs (001) substrate can direct the assembly of well defined arrangements of mounds during homoepitaxial growth, and that the sites at which mounds form on the template depend both on the growth temperature and on the deposited film thickness. We further find that a type of self-limiting behavior occurs in this system which can be exploited in selecting between different arrangements of mounds. Finally, and most interestingly from a technological standpoint, we find that an amplification of the spatial frequency of the mound arrangements, beyond that of the features of the template, is realizable in this system.

## 2. Results and Discussion

### 2.1. Directed Self-Assembly and Pattern Amplification

**Figure 1:**
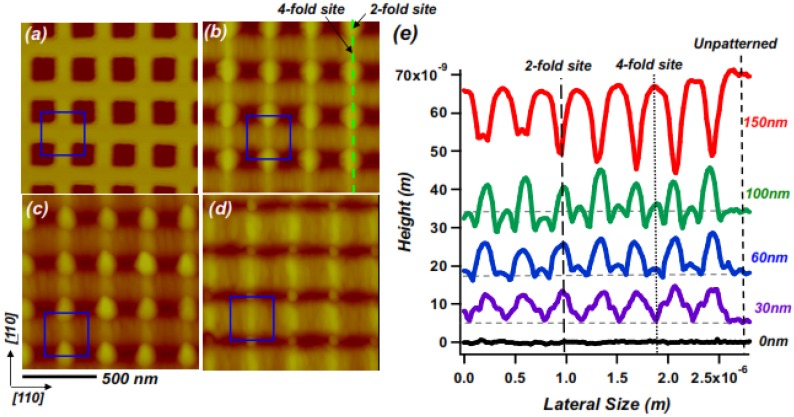
AFM images of nanopatterned GaAs (001) topography before and after homoepitaxial growth: (**a**) before growth; (**b**) after growth of 60 nm; (**c**) after growth of 100 nm; (**d**) after growth of 150 nm. Initial nanopit widths w =140 nm; center-center spacing between nanopits λ = 280 nm; growth temperature = 460 °C; growth rate = 0.28 nm/s; (**e**) Measured height profiles from Figure 1**a**–**d**, taken along [110] across bridge sites, as shown by green dashed line in (**b**); profiles are offset vertically for visibility. Blue squares show a pattern unit cell. The horizontal dashed lines indicate the height of the non-templated (“unpatterned”) regions of the surface.

Figure 1 shows the results of homoepitaxial growth on a patterned GaAs (001) substrate on which the initial width of each nanopit (measured between points of half maximum depth) was 140 nm. The starting topography is shown in Figure 1a, while subsequent panels show the topography which results from the growth of films of thickness (b) 60 nm; (c) 100 nm; and (d) 150 nm at a temperature of 460 °C. Figure 1b,c show the *directed assembly of mounds at particular sites on the template*. We refer to these as “2-fold bridge” sites, *i.e*., bridges between near-neighbor pits. This observation is in qualitative agreement with the predictions of our earlier kinetic Monte Carlo simulations [18] of growth at such sites on a patterned substrate within a particular temperature range, in the presence of an Ehrlich-Schwoebel barrier.

How does the template direct the mounds to assemble at particular sites? There can be multiple effects at play, depending on the energetics and kinetics of the mound formation. One likely contribution is topographical in origin. Mounds are unlikely to overhang the edge of a pit; this reduces the number of sites at which they can form, and leads to an *entropic* mound-pit edge interaction [18].

**Figure 2:**
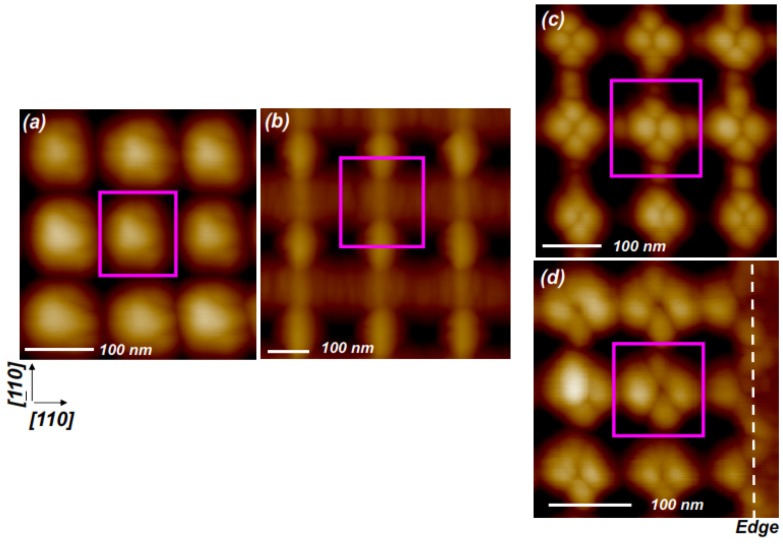
Temperature and lateral spatial periodicity (λ) dependence of directing self-assembly of mound structures (**a**) growth at 525 °C, λ = 120 nm; (**b**) growth at 460 °C, λ = 200 nm; (**c**,**d**) growth at 300 °C on nanopit-patterned surface with lateral spatial periodicities of 160 nm, and 120 nm, respectively. The dashed line in 2d marks the edge of the patterned region.

A second contributing effect comes from the competition of a “natural” mound spacing, which as we showed elsewhere [18] is determined by the growth temperature at a given set of fluxes, *versus* the spatial period artificially imposed by the template. (The temperature dependence could stem from the smaller mound capture zone for adatoms at lower temperatures, due to the shorter distances the adatoms can diffuse; this would impede mounds from coalescing to form larger ones.) The underlying physics behind the competition is reminiscent of the Frenkel-Kontorova model [19,20], which can lead to a series of structures with spatial frequencies whose magnitudes relative to those of the template can be changed [18,20], and possibly amplified [9,10,11,12,18]. Indeed our results are consistent with such a model. Figure 2 shows AFM images of the topography resulting from growth at a series of temperatures, and template spatial period. Strikingly, *the mound spatial frequency is amplified relative to that of the pattern*, resulting in an increased number of mounds per pattern unit cell as the growth temperature is lowered (Figure 2b–d) [21].

Further evidence for a competition between the natural mound spacing and the template period can be seen by comparing Figure 2c,d, where at a fixed growth temperature of 300 °C the number of mounds across a template unit cell decreases as the spatial period of the template is lowered. In these images the mound arrangements do not show a simple (*n* × *m*) periodicity; for example, in Figure 2c the mound positions can be referenced to a (a/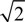 × a/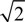)R45 unit cell with a basis of 5 mounds at (0,0), (A/3, B/3), (2A/3, B/3), (A/3, 2B/3), (2A/3, 2B/3), where A and B are the unit vectors of the lattice, *i.e*., A = (a/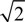, −a/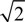) and B = (a/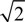, a/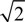). These observations are consistent with the results of our kMC simulations [18], which predict an increase in the “natural” mound size with temperature for growth on unpatterned surfaces.

An additional effect relevant in determining the arrangement of mounds can come from a tendency to nucleate at pit edges of certain orientations, if the edges of the pits provide heterogeneous nucleation sites [18] by locally reducing the perimeter energy, e.g., through a multistep reconstruction. We note that GaAs (001) is a 2-fold, rather than 4-fold symmetric surface, and the inequivalence of [110] and [110] step edges [22] seemingly explains the preference for one type of 2-fold bridge site observed in Figure 1b,c.

### 2.2. Self-Limiting Behavior

**Figure 3:**
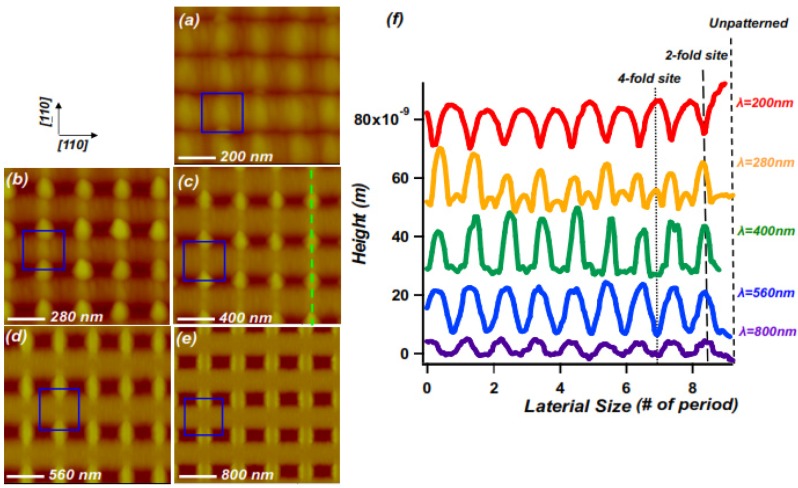
AFM images of nanopatterned GaAs (001) topography after homoepitaxial growth of 100 nm layer on nanopit arrays with different center to center spacings, λ; (**a**) λ = 200 nm; (**b**) λ = 280 nm; (**c**) λ = 400 nm; (**d**) λ = 560 nm; (**e**) λ = 800 nm; (**f**) Measured height profiles from Figure 3**a**–**e**, taken along [110], across bridge sites, as shown by green dashed line in (**c**); profiles are offset vertically for visibility; the horizontal axes are each normalized to the individual λ. Growth conditions as in Figure 1, *i.e*., growth temperature = 460 °C; growth rate = 0.28 nm/s.

We now consider one last effect, which leads to changes in the spatial arrangement of mound arrangements as growth continues. Figure 1e illustrates the evolution of the mounds during growth at 460 °C. It consists of a series of height profiles along the path corresponding to the dashed line in Figure 1b. After deposition of an overall film thickness of somewhere between 100 nm and 150 nm, a noticeable change in the evolution of the surface morphology occurs. While initially the mounds at 2-fold bridge sites grow and sharpen, after this point they evidently stop growing: their heights self-limit. Growing beyond the corresponding film thicknesses causes the mound heights to decrease [23]. In addition, beyond this point their heights are surpassed by those of mounds at “4-fold bridge” sites, *i.e*., at positions centered between quartets of neighboring nanopits. The observation that the film thickness at which the height of a mound self-limits depends on the type of site indicates that the mound arrangement can be tuned via controlling the amount of growth. We also find that the onset of self limiting behavior depends on the spatial period (λ) of the pattern. Figure 3 illustrates the lateral template length scale dependence of the mound heights after 100 nm of growth. For ease of comparison the lateral dimensions of the height profiles shown in Figure 3e are normalized to the period of each nanopit template. For the larger template spatial periods the surface evolves more slowly, and mounds at 2-fold bridge sites show a maximum height for *i.e*., λ ≈ 400 nm. For the array with a spatial period of 280 nm mounds at these sites are clearly shorter, while at the λ = 200 nm mounds at the 2-fold bridge sites have completely disappeared, and been replaced by downward cusps between the newly dominant mounds at 4-fold bridge sites.

**Figure 4:**
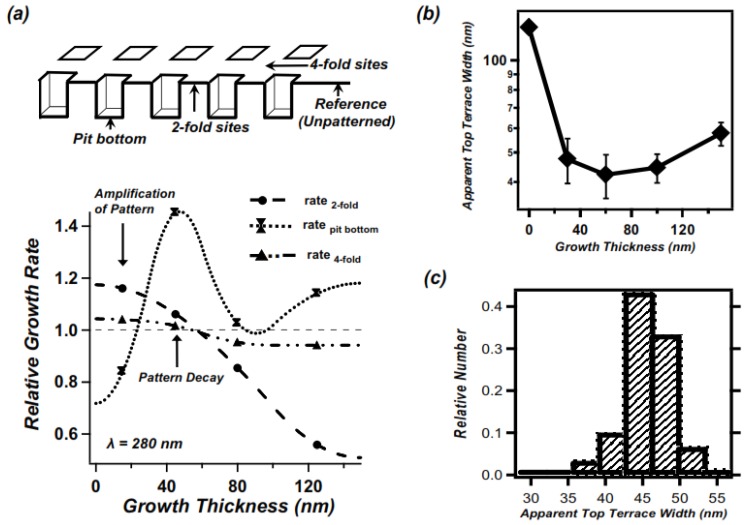
Growth rates and characteristics of pit array with λ = 280 nm (**a**) (top) Schematic showing positions referred to in growth rate analysis; (bottom) Growth rate, relative to level of unpatterned region of surface of mounds at 2-fold bridge sites (dashed line), mounds at 4-fold bridge sites (double dot-dashed line), nanopit bottoms (dotted line); markers show measured average rates, continuous curves are smooth interpolations, intended as a guide to the eye; (**b**) Measured widths of topmost terrace width for mounds at 2-fold bridge sites, measured along [110]; (**c**) Histogram of measured topmost terrace widths for a range of pattern periods measured at the growth thicknesses corresponding to the minimum.

We next show that the self-limiting behavior of mound heights is relevant to understanding the transient amplification of the pattern corrugation (height difference between peaks and valleys) during growth that we reported on earlier [17,24,25,26]. In Figure 4a we plot the growth rates of the heights of three different features, measured relative to the height of the surrounding unpatterned surface, and normalized to the average growth rate. The individual curves are for the mounds at 2-fold bridge sites (dashed curve), mounds at 4-fold bridge sites (dashed-dotted curve) and the pit bottoms (dotted curve). Using the unpatterned region as a reference level reduces the apparent difference in the range of growth over which mounds in the two types of sites dominate the topography, as shown in the supporting documents [23]. Most significantly, early on (*i.e*., for the smallest film thicknesses studied) the local growth rate at mound sites is greater than the average growth rate, while that at pit bottoms is below the average. This difference leads to an initial amplification of pattern corrugation during the early stage of growth. By a film thickness of 60 nm the self-limiting behavior of the mounds initiates, with the local mound growth rate falling behind both the average and that at pit bottoms. Indeed, coincident with this, the growth rate at the pit bottoms reaches a maximum, which well exceeds the average rate of growth. The pattern corrugation amplitude in this regime decays, consistent with our earlier reports [17,24,25,26]. The different rates of growth within and outside the pattern are consistent with an island formation (rather than step flow) mode, a large Ga adatom diffusion length at these temperatures [27], and, as we discuss below an island nucleation probability which depends on the local terrace width [17,28]. These observations also strongly suggest that the self-limiting growth of mounds is at least in part responsible for the transient nature of the amplification of the pattern corrugation.

A remaining question is: what physical mechanism lies behind the self-limiting growth behavior? Previously it has been suggested [16] that mound sidewall orientations should reach a steady state value; indeed we find that coincident with the initiation of self-limiting behavior mound sidewalls along the [110] azimuth evolve to orientations approximately 26° from (001), corresponding to {012}-type facets [29]. While perhaps significant, this faceting alone does not obviously explain the cessation of the growth of the mounds. A plausible explanation, based on observations that the mounds sharpen before self-limiting, is the existence of a minimum top terrace width, beneath which further islands do not nucleate atop the mounds. In Figure 4b, we plot the apparent top terrace width as a function of growth thickness based on height profiles across 2-fold bridge sites measured from Figure 1. The analysis shows a minimum size after growth of 60nm, *i.e*. coincident with the initiation of self-limiting behavior. Figure 4c shows a histogram of apparent apex terrace widths at the minimum shown in Figure 4b. It exhibits a distribution of widths, with an apparent peak value of 45–50 nm for the critical terrace size. This sets an upper limit for the critical width, as this measured value includes the convolution with a fairly blunt AFM probe. Deconvolution of the point spread function, using the manufacturer’s range of tip radii of 20 ± 10 nm would yield a value of 23 ± 23 nm, a range which includes a width as small as a single unit cell.

Intuitively one might expect the critical terrace width to be small, perhaps on the order of a unit cell of the GaAs (001)–c(4 × 4) reconstruction. A plausible hypothesis is that an effect related to “reaction limited island nucleation” of compound semiconductors during MBE growth, proposed by Kratzer and Scheffler, is responsible [30,31]. Specifically, incorporation of a new layer of GaAs into the solid would be prevented once the top terrace width is too small to have a finite probability for island nucleation to occur. Island nucleation involves multiple species (Ga adatoms, As_2_ molecules) adsorbed in sequence, along with selection of sufficiently strong absorption sites and surface geometry. A second possible mechanism, based on that proposed by Giesen *et al*. [32], is that the ES barrier vanishes due to quantum confinement effects of electronic states on the surface if the top terrace width drops below a certain critical size. The vanishing of the ES barrier at the apexes of mounds would increase the probability of interlayer mass transport from the top of the mounds to the pit bottoms, reducing the probability of island nucleation growth at the apexes, and initiating self-limiting behavior. Distinguishing between these and other possibilities is beyond the scope of this article.

## 3. Experimental Section

To create the templates used in this study we patterned GaAs (001) wafers using electron beam lithography followed by inductively-coupled plasma etching, creating several sets of square nanopit arrays in which the initial widths were varied systematically from 60 nm to 400 nm. The center-to-center spacings were held fixed at twice the initial nanopit widths, and the initial depths were held at approximately 50 nm. The patterned samples were cycled between a molecular beam epitaxy (MBE) growth chamber (VG V80H, Oxford Instruments, Abingdon, Oxford, UK) (base pressure 2 × 10^−11^ mbar) for homoepitaxial growth and an atomic force microscope (AFM) (DI 3100, Veeco/Bruker, Billerica, MA, USA) for surface topography characterization in atmosphere. The latter was operated in tapping mode with carbon nanotube-terminated probes, whose terminal radii were nominally between 10 nm and 30 nm. Before each growth experiment the surface oxide was desorbed by heating to 400 °C in the presence of atomic hydrogen, resulting in negligible desorption induced roughness. To track the evolution of individual features after various stages of growth, we used AFM in atmosphere to measure the topography of the surface. We then reintroduced the sample into the MBE chamber, repeated the deoxidation, and grew additional GaAs. An advantage of the pattern is that it allowed us to navigate back to the same features with the AFM after each growth step. The growth rate was held fixed at 0.28 nm/s with the As_2_ and Ga fluxes set for a beam equivalent pressure ratio of 10:1. The substrate temperature was determined by optical pyrometry with an emissivity correction that is calibrated using the thermal desorption temperature of the native GaAs oxide at 582 °C.

## 4. Conclusions

In conclusion, we have observed that it is possible to direct the assembly of arrangements of multilayer growth mounds on nanopatterned GaAs (001). Most significantly from a technological point of view we find that growth at low temperatures, near 300 °C leads to mound spatial frequencies exceeding those of the features of an artificially defined template, *i.e*., pattern amplification. We find that the spatial period of the arrangements can be changed by varying the growth temperature or pattern period, consistent with a competition between temperature-dependent, natural mound spacing and the spatial period of an artificially defined substrate topographical template. We also find that the film thickness over which the self-assembly of a particular mound assembly persists is finite. Once a mound reaches a self-limiting shape, it can only grow further via the apparently slow incorporation of atoms at steps which form its sidewalls. This is consistent with a critical, minimum terrace width for island nucleation. The self limitation of the mound heights casts new light on the origin of a transient amplification of an artificially imposed corrugation during homoepitaxy on GaAs (001). Finally, we note that the kinetic and entropic effects which dominate directed self assembly in this simple system must be taken into account in systems such as Ge on patterned Si (001) and InAs on patterned GaAs (001) along with the strain energy effects and interface tension effects which have been previously considered.

## Supplementary Materials

Supplementary File 1
