# Supplementary material for: Directed Kinetic Self-Assembly of Mounds on Patterned GaAs (001): Tunable Arrangement, Pattern Amplification and Self-Limiting Growth
Source: Nanomaterials (Basel). 2014 May 12;4(2):344–54. doi: 10.3390/nano4020344 (PMC5304676; doi:10.3390/nano4020344)
Supplement: Supplementary File 1 [file nanomaterials-04-00344-s001.pdf]

Supplementary Materials

Figure S1. Mound evolution for array with spatial period λ = 280 nm. (**a**) Mound heights *vs*. grown thickness, measured above local bridges on which they form; (closed triangles) mounds at 2-fold bridge sites; (closed squares) mounds at 4-fold bridge sites; (**b**) Mound heights vs. grown thickness, measured above the unpatterned part of the surface; (open triangles) mounds at 2-fold bridge sites; (open squares) mounds at 4-fold bridge sites; (**c**) AFM images showing entire nanopatterned array with different growth thickness; (**d**) Measured height profiles from Figure S1(**c**), taken along [110], across pit centers, as shown by red dashed line in Figure S1(**c**).

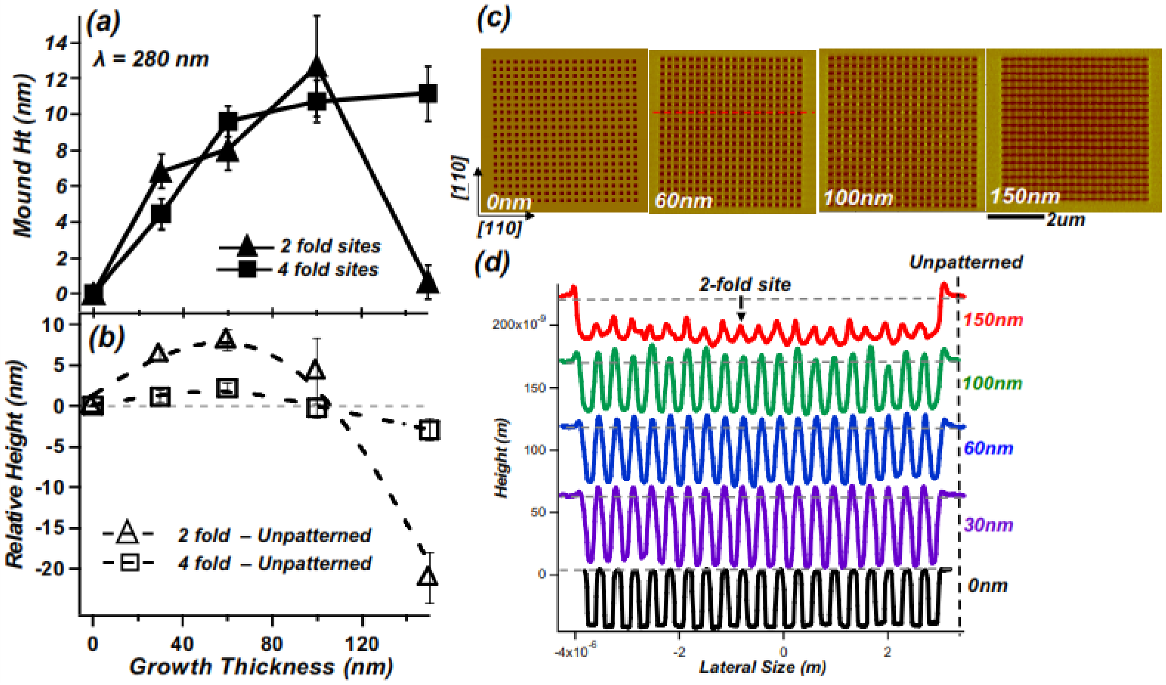

Figure S2. (**a**) SEM image of Directed Self Assembly mounds (at 2-fold sites) on patterned GaAs (001) surface. This image was taken after 150 nm of growth on pit array with λ = 400 nm. (**b**) Sidewall angle analysis of mounds as a function of growth thickness along [110] (green) and [$\underline{1}$10] (red) crystal orientations. The dashed line indicates the thickness that the self-limiting behavior occurs.

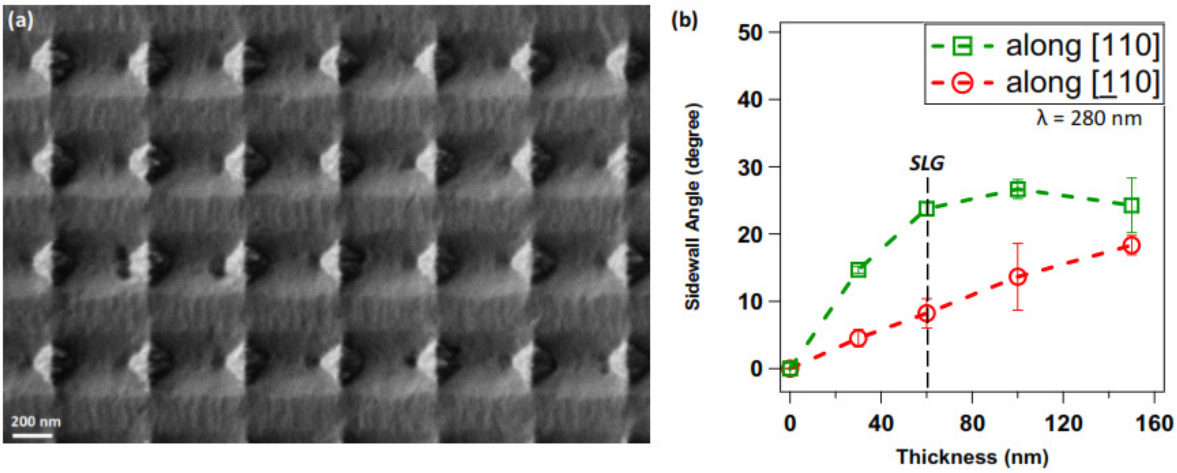

Figure S3. Lateral spatial periodicity (λ) dependence of directing self-assembly of mound formation for growth at 300 °C with (**a**) λ = 200 nm; (**b**) λ = 280 nm; (**c**) λ = 400 nm, and (**d**) λ = 800 nm. In agreement with our previous report [17,24,25] the tendency to nucleate mounds at [110]-oriented pit edges, combined with the limited natural size of the mounds at this temperature results in multiple mounds across parallel edges, but no obvious mounds at the interiors of the 2-fold bridges for large separations.

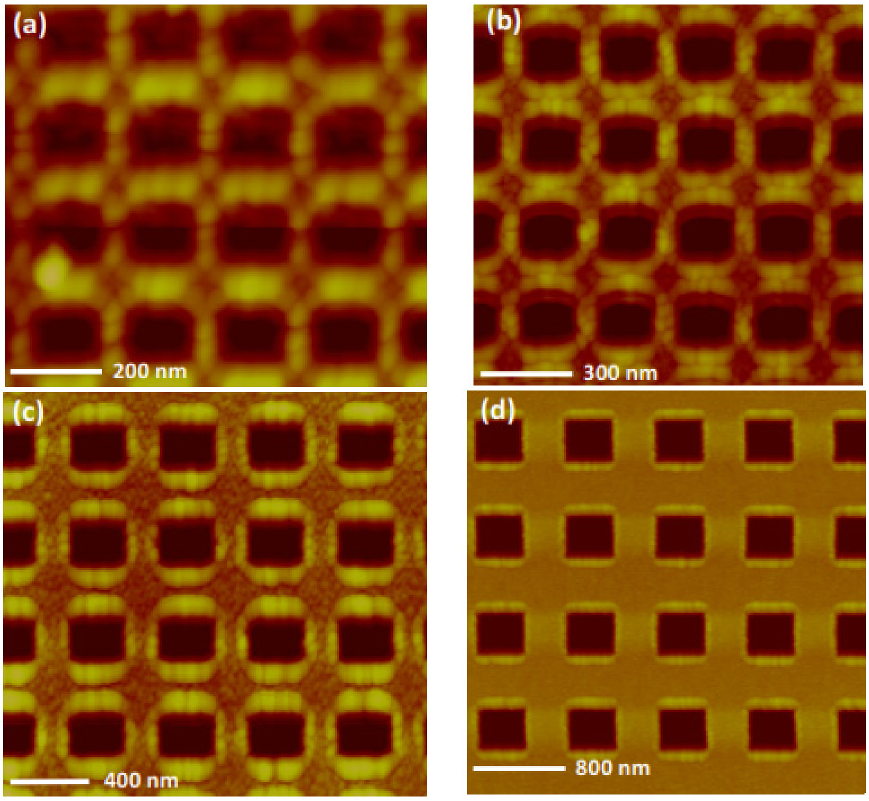

© 2014 by the authors; licensee MDPI, Basel, Switzerland. This article is an open access article distributed under the terms and conditions of the Creative Commons Attribution license (http://creativecommons.org/licenses/by/3.0/).
